# Supplementary material for: Aneurysmal subarachnoid hemorrhage in pregnancy: National trends of treatment, predictors, and outcomes
Source: PLoS One. 2023 May 4;18(5):e0285082. doi: 10.1371/journal.pone.0285082 (PMC10159186; doi:10.1371/journal.pone.0285082)
Supplement: S1 Table — (DOCX) [file pone.0285082.s001.docx]

Supplementary Table- ICD9 and ICD 10 diagnostic and procedure codes used to extract the study population and the subgroups from NIS.

|  | ICD 9 code | ICD 10 code |
| --- | --- | --- |
| SAH | 430,431,4329 | 'I60','I629', 'I61' |
| CLIP | 3951 | ('03VG0CZ',  '03VG3CZ',  '03VG4CZ',  '03VH0CZ',  '03VH3CZ',  '03VH4CZ',  '03VJ0CZ',  '03VJ3CZ',  '03VJ4CZ',  '03VK0CZ',  '03VK3CZ',  '03VK4CZ',  '03VL0CZ',  '03VL3CZ',  '03VL4CZ',  '03VP0CZ',  '03VP3CZ',  '03VP4CZ',  '03VQ0CZ',  '03VQ3CZ',  '03VQ4CZ' |
| COIL | 3972, 3975, 3976,3979 | ('03LG3',  '03LH3',  '03LJ3',  '03LK3',  '03LL3',  '03LM3',  '03LN3',  '03LP3',  '03LQ3',  '03LG3',  '03LH3',  '03LJ3',  '03LK3',  '03LL3',  '03LM3',  '03LN3',  '03LP3',  '03LQ3',  '03BG3',  '03BH3',  '03BJ3',  '03BK3',  '03BL3',  '03BP3',  '03BQ3',  '00B73',  '00B03',  '00BC3',  '00503',  '00573',  '005C3',  '03VG3',  '03VH3',  '03VJ3',  '03VK3',  '03VL3',  '03VM3',  '03VN3',  '03VP3',  '03VQ3',  '05BL3',  '00C73',  '00C03',  '00CC3',  '035G3',  '035H3',  '035J3',  '035K3',  '035L3',  '035M3',  '035N3',  '035Q3',  '035P3',  '05LL3',  '05LM3',  '05LN3',  '05LR3',  '05LS3',  '00973',  '009C3',  '00903',  '03CG3',  '03CH3',  '03CJ3',  '03CK3',  '03CL3',  '03CP3',  '03CQ3',  '03HG3',  '03HP3',  '03HQ3',  '03NG3',  '03UG3',  '03QG3',  '039G3') |
| TBI | 800,801,803,804,850,  851,852,853,854,873 | ('S00','S01','S02','S03',  'S04','S05','S06','S07'  ,'S08','S09') |
| AVM | 74781 | ('Q282', 'Q283' |
| Brain tumor | 1910,1911,1912,1913,1914,1915  ,1917,1918,1919,1920,1921,1983,  2250,2251,2252,2371,2375,2376,2396, 2397, V1085,V1086 | ('C710','C711','C712',  'C713','C714','C715','C716',  'C717','C718','C719','C7250',  'C700','C7931','D332','D333',  'D320','D334','D445','D432',  'D420','D496','D497','Z85841',  'Z85848') |
| Pregnancy Dx | ("63540" , "63541" , "63542" , "63550" , "63551" , "63552" , "63560" , "63561" ,  "63562" , "63570" , "63571" , "63572" , "63580" , "63581" , "63582" , "63590" ,  "63591" , "63592" , "63600" , "63601" ,  "63602" , "63610" , "63611" , "63612" , "63620" , "63621" , "63622" , "63630" ,  "63631" , "63632" , "63640" , "63641" , "63642" , "63650" , "63651" , "63652" ,  "63660" , "63661" , "63662" , "63670" , "63671" , "63672" , "63680" , "63681" ,  "63682" , "63690" , "63691" , "63692" , "63700" , "63701" , "63702" , "63710" ,  "63711" , "63712" , "63720" , "63721" , "63722" , "63730" , "63731" , "63732" ,  "63740" , "63741" , "63742" , "63750" , "63751" , "63752" , "63760" , "63761" ,  "63762" , "63770" , "63771" , "63772" , "63780" , "63781" , "63782" , "63790" ,  "63791" , "63792" , "6380 " , "6381 " , "6382 " , "6383 " , "6384 " , "6385 " ,  "6386 " , "6387 " , "6388 " , "6389 " , "6390 " , "6391 " , "6392 " , "6393 " ,  "6394 " , "6395 " , "6396 " , "6398" , "6399" , "64000" , "64001" , "64003" ,  "64080" , "64081" , "64083" , "64090" , "64091" , "64093" , "64100" , "64101" ,  "64103" , "64110" , "64111" , "64113" , "64120" , "64121" , "64123" , "64130" ,  "64131" , "64133" , "64180" , "64181" , "64183" , "64190" , "64191" , "64193" ,  "64200" , "64201" , "64202" , "64203" , "64204" , "64210" , "64211" , "64212" ,  "64213" , "64214" , "64220" , "64221" , "64222" , "64223" , "64224" , "64230" ,  "64231" , "64232" , "64233" , "64234" , "64240" , "64241" , "64242" , "64243" ,  "64244" , "64250" , "64251" , "64252" , "64253" , "64254" , "64260" , "64261" ,  "64262" , "64263" , "64264" , "64270" , "64271" , "64272" , "64273" , "64274" ,  "64290" , "64291" , "64292" , "64293" , "64294" , "64300" , "64301" , "64303" ,  "64310" , "64311" , "64313" , "64320" , "64321" , "64323" , "64380" , "64381" ,  "64383" , "64390" , "64391" , "64393" , "64400" , "64403" , "64410" , "64413" ,  "64420" , "64421" , "64500" , "64501" , "64503" , "64510" , "64511" , "64513" ,  "64520" , "64521" , "64523" , "64600" , "64601" , "64603" , "64610" , "64611" ,  "64612" , "64613" , "64614" , "64620" , "64621" , "64622" , "64623" , "64624" ,  "64630" , "64631" , "64633" , "64640" , "64641" , "64642" , "64643" , "64644" ,  "64650" , "64651" , "64652" , "64653" , "64654" , "64660" , "64661" , "64662" ,  "64663" , "64664" , "64670" , "64671" , "64673" , "64680" , "64681" , "64682" ,  "64683" , "64684" , "64690" , "64691" , "64693" , "64700" , "64701" , "64702" ,  "64703" , "64704" , "64710" , "64711" , "64712" , "64713" , "64714" , "64720" ,  "64721" , "64722" , "64723" , "64724" , "64730" , "64731" , "64732" , "64733" ,  "64734" , "64740" , "64741" , "64742" , "64743" , "64744" , "64750" , "64751" ,  "64752" , "64753" , "64754" , "64760" , "64761" , "64762" , "64763" , "64764" ,  "64780" , "64781" , "64782" , "64783" , "64784" , "64790" , "64791" , "64792" ,  "64793" , "64794" , "64800" , "64801" , "64802" , "64803" , "64804" , "64810" ,  "64811" , "64812" , "64813" , "64814" , "64820" , "64821" , "64822" , "64823" ,  "64824" , "64830" , "64831" , "64832" , "64833" , "64834" , "64840" , "64841" ,  "64842" , "64843" , "64844" , "64850" , "64851" , "64852" , "64853" , "64854" ,  "64860" , "64861" , "64862" , "64863" , "64864" , "64870" , "64871" , "64872" ,  "64873" , "64874" , "64880" , "64881" , "64882" , "64883" , "64884" , "64890" ,  "64891" , "64892" , "64893" , "64894" , "64900" , "64901" , "64902" , "64903" ,  "64904" , "64910" , "64911" , "64912" , "64913" , "64914" , "64920" , "64921" ,  "64922" , "64923" , "64924" , "64930" , "64931" , "64932" , "64933" , "64934" ,  "64940" , "64941" , "64942" , "64943" , "64944" , "64950" , "64951" , "64953" ,  "64960" , "64961" , "64962" , "64963" , "64964" , "64970" , "64971" , "64973" ,  "64981" , "64982" , "650 " , "65100" , "65101" , "65103" , "65110" , "65111" ,  "65113" , "65120" , "65121" , "65123" , "65130" , "65131" , "65133" , "65140" ,  "65141" , "65143" , "65150" , "65151" , "65153" , "65160" , "65161" , "65163" ,  "65170" , "65171" , "65173" , "65180" , "65181" , "65183" , "65190" , "65191" ,  "65193" , "65200" , "65201" , "65203" , "65210" , "65211" , "65213" , "65220" ,  "65221" , "65223" , "65230" , "65231" , "65233" , "65240" , "65241" , "65243" ,  "65250" , "65251" , "65253" , "65260" , "65261" , "65263" , "65270" , "65271" ,  "65273" , "65280" , "65281" , "65283" , "65290" , "65291" , "65293" , "65300" ,  "65301" , "65303" , "65310" , "65311" , "65313" , "65320" , "65321" , "65323" ,  "65330" , "65331" , "65333" , "65340" , "65341" , "65343" , "65350" , "65351" ,  "65353" , "65360" , "65361" , "65363" , "65370" , "65371" , "65373" , "65380" ,  "65381" , "65383" , "65390" , "65391" , "65393" , "65400" , "65401" , "65402" ,  "65403" , "65404" , "65410" , "65411" , "65412" , "65413" , "65414" , "65420" ,  "65421" , "65423" , "65430" , "65431" , "65432" , "65433" , "65434" , "65440" ,  "65441" , "65442" , "65443" , "65444" , "65450" , "65451" , "65452" , "65453" ,  "65454" , "65460" , "65461" , "65462" , "65463" , "65464" , "65470" , "65471" ,  "65472" , "65473" , "65474" , "65480" , "65481" , "65482" , "65483" , "65484" ,  "65490" , "65491" , "65492" , "65493" , "65494" , "65500" , "65501" , "65503" ,  "65510" , "65511" , "65513" , "65520" , "65521" , "65523" , "65530" , "65531" ,  "65533" , "65540" , "65541" , "65543" , "65550" , "65551" , "65553" , "65560" ,  "65561" , "65563" , "65570" , "65571" , "65573" , "65580" , "65581" , "65583" ,  "65590" , "65591" , "65593" , "65600" , "65601" , "65603" , "65610" , "65611" ,  "65613" , "65620" , "65621" , "65623" , "65630" , "65631" , "65633" , "65640" ,  "65641" , "65643" , "65650" , "65651" , "65653" , "65660" , "65661" , "65663" ,  "65670" , "65671" , "65673" , "65680" , "65681" , "65683" , "65690" , "65691" ,  "65693" , "65700" , "65701" , "65703" , "65800" , "65801" , "65803" , "65810" ,  "65811" , "65813" , "65820" , "65821" , "65823" , "65830" , "65831" , "65833" ,  "65840" , "65841" , "65843" , "65880" , "65881" , "65883" , "65890" , "65891" ,  "65893" , "65900" , "65901" , "65903" , "65910" , "65911" , "65913" , "65920" ,  "65921" , "65923" , "65930" , "65931" , "65933" , "65940" , "65941" , "65943" ,  "65950" , "65951" , "65953" , "65960" , "65961" , "65963" , "65970" , "65971" ,  "65973" , "65980" , "65981" , "65983" , "65990" , "65991" , "65993" , "66000" ,  "66001" , "66003" , "66010" , "66011" , "66013" , "66020" , "66021" , "66023" ,  "66030" , "66031" , "66033" , "66040" , "66041" , "66043" , "66050" , "66051" ,  "66053" , "66060" , "66061" , "66063" , "66070" , "66071" , "66073" , "66080" ,  "66081" , "66083" , "66090" , "66091" , "66093" , "66100" , "66101" , "66103" ,  "66110" , "66111" , "66113" , "66120" , "66121" , "66123" , "66130" , "66131" ,  "66133" , "66140" , "66141" , "66143" , "66190" , "66191" , "66193" , "66200" ,  "66201" , "66203" , "66210" , "66211" , "66213" , "66220" , "66221" , "66223" ,  "66230" , "66231" , "66233" , "66300" , "66301" , "66303" , "66310" , "66311" ,  "66313" , "66320" , "66321" , "66323" , "66330" , "66331" , "66333" , "66340" ,  "66341" , "66343" , "66350" , "66351" , "66353" , "66360" , "66361" , "66363" ,  "66380" , "66381" , "66383" , "66390" , "66391" , "66393" , "66400" , "66401" ,  "66404" , "66410" , "66411" , "66414" , "66420" , "66421" , "66424" , "66430" ,  "66431" , "66434" , "66440" , "66441" , "66444" , "66450" , "66451" , "66454" ,  "66460" , "66461" , "66464" , "66480" , "66481" , "66484" , "66490" , "66491" ,  "66494" , "66500" , "66501" , "66503" , "66510" , "66511" , "66512" , "66514" ,  "66520" , "66522" , "66524" , "66530" , "66531" , "66534" , "66540" , "66541" ,  "66544" , "66550" , "66551" , "66554" , "66560" , "66561" , "66564" , "66570" ,  "66571" , "66572" , "66574" , "66580" , "66581" , "66582" , "66583" , "66584" ,  "66590" , "66591" , "66592" , "66593" , "66594" , "66600" , "66602" , "66604" ,  "66610" , "66612" , "66614" , "66620" , "66622" , "66624" , "66630" , "66632" ,  "66634" , "66700" , "66702" , "66704" , "66710" , "66712" , "66714" , "66800" ,  "66801" , "66802" , "66803" , "66804" , "66810" , "66811" , "66812" , "66813" ,  "66814" , "66820" , "66821" , "66822" , "66823" , "66824" , "66880" , "66881" ,  "66882" , "66883" , "66884" , "66890" , "66891" , "66892" , "66893" , "66894" ,  "66900" , "66901" , "66902" , "66903" , "66904" , "66910" , "66911" , "66912" ,  "66913" , "66914" , "66920" , "66921" , "66922" , "66923" , "66924" , "66930" ,  "66932" , "66934" , "66940" , "66941" , "66942" , "66943" , "66944" , "66950" ,  "66951" , "66960" , "66961" , "66970" , "66971" , "66980" , "66981" , "66982" ,  "66983" , "66984" , "66990" , "66991" , "66992" , "66993" , "66994" , "67000" ,  "67002" , "67004" , "67010" , "67012" , "67014" , "67020" , "67022" , "67024" ,  "67030" , "67032" , "67034" , "67080" , "67082" , "67084" , "67100" , "67101" ,  "67102" , "67103" , "67104" , "67110" , "67111" , "67112" , "67113" , "67114" ,  "67120" , "67121" , "67122" , "67123" , "67124" , "67130" , "67131" , "67133" ,  "67140" , "67142" , "67144" , "67150" , "67151" , "67152" , "67153" , "67154" ,  "67180" , "67181" , "67182" , "67183" , "67184" , "67190" , "67191" , "67192" ,  "67193" , "67194" , "67200" , "67202" , "67204" , "67300" , "67301" , "67302" ,  "67303" , "67304" , "67310" , "67311" , "67312" , "67313" , "67314" , "67320" ,  "67321" , "67322" , "67323" , "67324" , "67330" , "67331" , "67332" , "67333" ,  "67334" , "67380" , "67381" , "67382" , "67383" , "67384" , "67400" , "67401" ,  "67402" , "67403" , "67404" , "67410" , "67412" , "67414" , "67420" , "67422" ,  "67424" , "67430" , "67432" , "67434" , "67440" , "67442" , "67444" , "67450" ,  "67451" , "67452" , "67453" , "67454" , "67480" , "67482" , "67484" , "67490" ,  "67492" , "67494" , "67500" , "67501" , "67502" , "67503" , "67504" , "67510" ,  "67511" , "67512" , "67513" , "67514" , "67520" , "67521" , "67522" , "67523" ,  "67524" , "67580" , "67581" , "67582" , "67583" , "67584" , "67590" , "67591" ,  "67592" , "67593" , "67594" , "67600" , "67601" , "67602" , "67603" , "67604" ,  "67610" , "67611" , "67612" , "67613" , "67614" , "67620" , "67621" , "67622" ,  "67623" , "67624" , "67630" , "67631" , "67632" , "67633" , "67634" , "67640" ,  "67641" , "67642" , "67643" , "67644" , "67650" , "67651" , "67652" , "67653" ,  "67654" , "67660" , "67661" , "67662" , "67663" , "67664" , "67680" , "67681" ,  "67682" , "67683" , "67684" , "67690" , "67691" , "67692" , "67693" , "67694" ,  "677 " , "67800" , "67801" , "67803" , "67810" , "67811" , "67813" , "67900" ,  "67901" , "67902" , "67903" , "67904" , "67910" , "67911" , "67912" , "67913" ,  "67914" , "7923 " , "V220 " , "V221 " , "V222 " , "V230 " , "V231 " , "V232 " ,  "V233 " , "V234 " , "V2341" , "V2342" , "V2349" , "V235 " , "V237 " , "V238 " ,  "V2381" , "V2382" , "V2383" , "V2384" , "V2385" , "V2386" , "V2387" , "V2389" ,  "V239 " , "V240 " , "V241 " , "V242 " , "V280 " , "V281" , "V282 " , "V283 " ,  "V284 " , "V285 " , "V286 " , "V288 " , "V2881" , "V2882" , "V2889" , "V289 " ,  "6901" , "6902" , "7491", "720", "721 " , "7221" , "7229" , "7231" , "7239" , "724 " , "7251" , "7252" ,  "7253" , "7254" , "726 " , "7271" , "7279" , "728 " , "729 " , "7301" , "7309" ,  "731 " , "7321" , "7322" , "733 " , "734 " , "7351" , "7359" , "736 " , "738 " ,  "7391" , "7392" , "7393" , "7394" , "7399" , "740 " , "741 " , "742 " , "743 " ,  "744 " , "7491" , "7499" , "750 " , "751 " , "752 " , "7531" , "7532" , "7533" ,  "7534" , "7535" , "7536" , "7537" , "7538" , "754 " , "7550" , "7551" , "7552" ,  "7561" , "7562" , "7569", "757 " "758 " , "7591" , "7592" , "7593" , "7594" ,  "7599" | I10_Dx  ('O00',  'O01',  'O02',  'O03',  'O04',  'O07',  'O08',  'O09',  'O10',  'O11',  'O12',  'O13',  'O14',  'O15',  'O16',  'O20',  'O21',  'O22',  'O23',  'O24',  'O25',  'O26',  'O28',  'O29',  'O30',  'O31',  'O32',  'O33',  'O34',  'O35',  'O36',  'O40',  'O41',  'O42',  'O43',  'O44',  'O45',  'O46',  'O47',  'O48',  'O60',  'O61',  'O62',  'O63',  'O64',  'O65',  'O66',  'O67',  'O68',  'O69',  'O70',  'O71',  'O72',  'O73',  'O74',  'O75',  'O76',  'O77',  'O80',  'O82',  'O85',  'O86' ,  'O87',  'O88',  'O89 ',  'O90',  'O91',  'O92',  'O94',  'O98',  'O99',  'O9A',  'Z33',  'Z34',  'Z36',  'Z37',  'Z38',  'Z39',  'Z3A')  I10_PR  '102','109','10A','10D',  '10E','10H','10J','10P','10Q',  '10S','10T','10Y' |
| Pregnancy Procedure |  | '102','109','10A','10D',  '10E','10H','10J','  10P','10Q',  '10S','10T','10Y' |
| Hypertensive disease of pregnancy | ('6425','6427','6424','6426') | 'O14','O15','O11' |
| Stupor | 78009 | ('R401','R400') |
| Coma | ('78001','78003') | ('R402','R403') |
| Hydrocephalus | 3314 | ('G911', 'G918','G919','G913') |
| Paresis | ("4382", "4383", "4384", "4385", "342") | ("G81","G82","G83",  "I6905","I6915","I6925",  "I6935","I6985","I6995") |
| Aphasia | ("4381","7843") | ("R47","I69020","I69120",  "I69220","I69320","I69820",  "I69920") |
| Cranial nerve palsy | 3529,3526,3529,9518,9519,3785,37940, 37941, 37942, 37943 | ("G53","G527","G529","  S048","S049","H49","H5700",  "H5702","H5703","H5704") |
| EVD | 0139,022,023 | ('0090','0096','00C0','00C6',  '00H0','00H6','00P0','00P6',  '00W0','00W6','0076','00B6') |
| MV | 3199,9604,9671,9670 | ('0BH13EZ', '0BH17EZ', '0BH18EZ','5A193','5A194',  '5A195') |
