# Supplementary material for: Aneurysmal subarachnoid hemorrhage in pregnancy: National trends of treatment, predictors, and outcomes
Source: PLoS One. 2023 May 4;18(5):e0285082. doi: 10.1371/journal.pone.0285082 (PMC10159186; doi:10.1371/journal.pone.0285082)
Supplement: Attachment — Submitted filename: response to reviewers.docx [file pone.0285082.s002.docx]

Response to Reviewer #1:

Thank you for pointing out this limitation and the granular analysis. Using the break down of the population by age published from the year 2021 with estimating the total population to be 331.89 million, the proportion of the women between the ages of 18 through 45 was estimated to be 58million people which is likely a slight over estimation compared to this population between the years of 2012 to 2018 given the increase in population(1). De Rooj et al in their international systematic review estimated the incidence of aneurysmal subarachnoid hemorrhage for women between the ages of 18-45 to be around 7 per 100,000 person year.(2) Using this estimation the incidence of aSAH within the women in this age group over this time period would be estimated to be 32480. So the study cohort with 13351 patients is representative of less than 50% of the true cohort. This limitation will affect the generalizability of the conclusion to the general united states population. According changes were made to the manuscript to elaborate on this limitation.

Response to Reviewer #2:

Thank you for pointing out this limitation in inclusion criteria which has definitely confounded the selection and therefore the observed outcome. To ensure that the underlying etiology of subarachnoid hemorrhage in the included patients were aneurysmal we limited the inclusion criteria to the patients who have had open or endovascular surgical treatment. Based on the coding used including all subarachnoid patients specially in young women and young pregnant women would potentially include a large proportion of non-aneurysmal subarachnoid hemorrhages.

However, as it was suggested by not including patients with aneurysmal subarachnoid hemorrhage who did not undergo treatment could potentially exclude the patients who had devastating neurological disability after the ictus of hemorrhage. By excluding all or proportion of such patients could potentially observe high rate of good function of outcome and lower mortality rate as mentioned in the limitation paragraph of the discussion section.

As again suggested by the reviewer in the recently published pooled case series and systematic reviews show a higher rate of mortality compared to our observation (6.8%). Robba et al. in their pooled review of 52 patients 9% of whom did not receive any treatments with reported mortality rate of 11%. (3) Beighley et al in a more recent systemic review of 54 patients 22% whom did not undergo treatment report mortality rate of 20%. (4) According changes were made to the discussion section to further elaborate on this limitation.

Thank you for the comment about the power of the study. We agree that with a prospective study, power analysis would significantly help study design and conclusions. However, we are utilizing a retrospective cohort and because of the study design which demonstrates likely no difference in between the effect size for mortality and functional outcome between the studied cohort post hoc power calculation would not likely be helpful be helpful.(5-7) Specially given the post hoc power analysis is to identify that the “non-significant” hypothesis test failed to achieve significance as it was not powerful enough and requiring higher degrees of freedom and a larger sample size. Though the number of subjects remain limited in this study it is performed using one of the larger openly available database of patients in the united states. The largest pooled series and systemic review of aneurysmal subarachnoid hemorrhage in pregnancy have included upto 50 patients which shows the infrequency of such cases and difficulty with obtaining a larger sample size.

Response to Reviewer #3:

Thank you for pointing out the non specificity of the criteria for measurement of functional outcome. Given the limitations of use of NIS database discharge destination was the more reliable variable that we could utilize for comparison of these cohorts. According changes were made to the discussion section to emphasize this limitation.

Response to Reviewer #4:

Thank you for pointing out the limitation of NIS based analyses. As described in the discussion section and further pointed out by the other reviewers use of NIS has led to need for use of more strict inclusion criteria which affects the generalizability of the observations. Additionally given the limitations the measure used for evaluation of functional outcome is non specific and based only the condition at discharge with no long term outcome available. The manuscript to further emphasize such short comings.

1.	Duffin E. Resident population of the United States by sex and age as of July 1, 2021 statista.com2022 [

2.	de Rooij NK, Linn FH, van der Plas JA, Algra A, Rinkel GJ. Incidence of subarachnoid haemorrhage: a systematic review with emphasis on region, age, gender and time trends. J Neurol Neurosurg Psychiatry. 2007;78(12):1365-72.

3.	Robba C, Bacigaluppi S, Bragazzi NL, Bilotta F, Sekhon MS, Bertuetti R, et al. Aneurysmal Subarachnoid Hemorrhage in Pregnancy-Case Series, Review, and Pooled Data Analysis. World Neurosurg. 2016;88:383-98.

4.	Beighley A, Glynn R, Scullen T, Mathkour M, Werner C, Berry JF, et al. Aneurysmal subarachnoid hemorrhage during pregnancy: a comprehensive and systematic review of the literature. Neurosurg Rev. 2021;44(5):2511-22.

5.	Althouse AD. Post Hoc Power: Not Empowering, Just Misleading. J Surg Res. 2021;259:A3-A6.

6.	Goodman SN, Berlin JA. The use of predicted confidence intervals when planning experiments and the misuse of power when interpreting results. Ann Intern Med. 1994;121(3):200-6.

7.	Hoenig JM HD. The abuse of power: the pervasive fallacy of power calculations for data analysis. . Am Stat 2001;55:19-24.
